# Blockade of Gap Junction Hemichannel Suppresses Disease Progression in Mouse Models of Amyotrophic Lateral Sclerosis and Alzheimer's Disease

**DOI:** 10.1371/journal.pone.0021108

**Published:** 2011-06-21

**Authors:** Hideyuki Takeuchi, Hiroyuki Mizoguchi, Yukiko Doi, Shijie Jin, Mariko Noda, Jianfeng Liang, Hua Li, Yan Zhou, Rarami Mori, Satoko Yasuoka, Endong Li, Bijay Parajuli, Jun Kawanokuchi, Yoshifumi Sonobe, Jun Sato, Koji Yamanaka, Gen Sobue, Tetsuya Mizuno, Akio Suzumura

**Affiliations:** 1 Department of Neuroimmunology, Research Institute of Environmental Medicine, Nagoya University, Furo-cho, Chikusa-ku, Nagoya, Japan; 2 Futuristic Environmental Simulation Center, Research Institute of Environmental Medicine, Nagoya University, Furo-cho, Chikusa-ku, Nagoya, Japan; 3 Laboratory for Motor Neuron Disease, RIKEN Brain Science Institute, Hirosawa, Wako, Saitama, Japan; 4 Department of Neurology, Nagoya University Graduate School of Medicine, Tsurumai-cho, Showa-ku, Nagoya, Japan; Thomas Jefferson University, United States of America

## Abstract

**Background:**

Glutamate released by activated microglia induces excitotoxic neuronal death, which likely contributes to non-cell autonomous neuronal death in neurodegenerative diseases, including amyotrophic lateral sclerosis and Alzheimer's disease. Although both blockade of glutamate receptors and inhibition of microglial activation are the therapeutic candidates for these neurodegenerative diseases, glutamate receptor blockers also perturbed physiological and essential glutamate signals, and inhibitors of microglial activation suppressed both neurotoxic/neuroprotective roles of microglia and hardly affected disease progression. We previously demonstrated that activated microglia release a large amount of glutamate specifically through gap junction hemichannel. Hence, blockade of gap junction hemichannel may be potentially beneficial in treatment of neurodegenerative diseases.

**Methods and Findings:**

In this study, we generated a novel blood-brain barrier permeable gap junction hemichannel blocker based on glycyrrhetinic acid. We found that pharmacologic blockade of gap junction hemichannel inhibited excessive glutamate release from activated microglia *in vitro* and *in vivo* without producing notable toxicity. Blocking gap junction hemichannel significantly suppressed neuronal loss of the spinal cord and extended survival in transgenic mice carrying human superoxide dismutase 1 with G93A or G37R mutation as an amyotrophic lateral sclerosis mouse model. Moreover, blockade of gap junction hemichannel also significantly improved memory impairments without altering amyloid β deposition in double transgenic mice expressing human amyloid precursor protein with K595N and M596L mutations and presenilin 1 with A264E mutation as an Alzheimer's disease mouse model.

**Conclusions:**

Our results suggest that gap junction hemichannel blockers may represent a new therapeutic strategy to target neurotoxic microglia specifically and prevent microglia-mediated neuronal death in various neurodegenerative diseases.

## Introduction

Microglia are macrophage-like resident immune cells of the central nervous system (CNS). They function as not only antigen-presenting cells but also effector cells that have been shown to damage neural cells directly *in vitro* and *in vivo* [1]. Microgliosis—*i.e.*, the accumulation of activated microglia—is a pathologic hallmark of various neurologic disorders, including trauma, ischemia, inflammation, epilepsy, and such neurodegenerative diseases as multiple sclerosis, Huntington's disease, Parkinson's disease, amyotrophic lateral sclerosis (ALS), and Alzheimer's disease (AD) [2]–[4]. Activated microglia release large amounts of glutamate and induce excitotoxicity via N-methyl-D-aspartate (NMDA) receptor signaling [5]–[8]. NMDA receptor signaling increases Ca^2+^ influx, resulting in Ca^2+^/calmodulin-dependent protein kinase (CaMK) activation. CaMK augments nitric oxide (NO) production by activating neuronal NO synthase. NO inhibits mitochondrial respiratory chain complex IV activity and induces a rapid drop in intracellular ATP levels, which negatively affects dendritic and axonal transport. Impaired transport causes cytoskeletal and motor protein accumulation at sites of neuritic beading [6]. This energy-starved condition represents neuronal dysfunction state and eventually leads to neuronal death (recently termed non-cell-autonomous neuronal death [9]). This process has been postulated as a major cause of neuronal damage in many neurologic diseases [10], [11]. Thus, blocking glutamate receptors has emerged as a potential therapeutic approach for several neurodegenerative diseases, however, associated perturbations in physiologic glutamate signaling lead to severe adverse effects [12]. Whereas inhibition of microglial activation has also been considered as a therapeutic strategy, these cells play neuroprotective roles that are mediated by release of neurotrophic factors, glutamate uptake, and sequestering of neurotoxic substances [2], [13]–[16]. After all, these clinical trials have largely failed in the last two decades [17]–[19]. Therefore, efforts are underway to target neurotoxic microglia specifically.

Gap junctions are composed of two adjacent hemichannels, which directly connect the cytoplasmic compartments of adjacent cells and allow small molecules (<1 kD) and ions to pass freely between cells [20]. Recent evidence suggests that “free” hemichannels on the cell surface play an important role as communication channels between the cytosol and extracellular milieu [20]. We recently showed that gap junction hemichannels are the main avenue of excessive glutamate release from neurotoxic activated microglia [5]. Moreover, we demonstrated that blockade of gap junction hemichannels by glycyrrhetinic acid derivatives significantly prevented activated microglia/macrophage-mediated neuronal death *in vitro* [5], [21], [22] and *in vivo* using rodent models of transient ischemic brain injury [23] and experimental autoimmune encephalomyelitis [24], which are associated with the blood-brain barrier (BBB) damage. In the present study, we investigated whether a gap junction hemichannel blocker alleviates neurodegeneration in mouse models of ALS and AD, two representative neurodegenerative diseases that are thought to involve pathologic microglial responses. Unfortunately, glycyrrhetinic acid and its derivatives including carbenoxolone hardly penetrate intact BBB observed in ALS and AD. Therefore we generated a novel BBB permeable gap junction hemichannel blocker based on glycyrrhetinic acid. Our findings suggest that blockade of gap junction hemichannels may be a potent therapeutic strategy to counteract microglia-induced excitotoxicity in neurodegenerative diseases.

## Results

### Effects of INI-0602 on microglial glutamate release *in vitro* and *in vivo*

**Figure 1:**
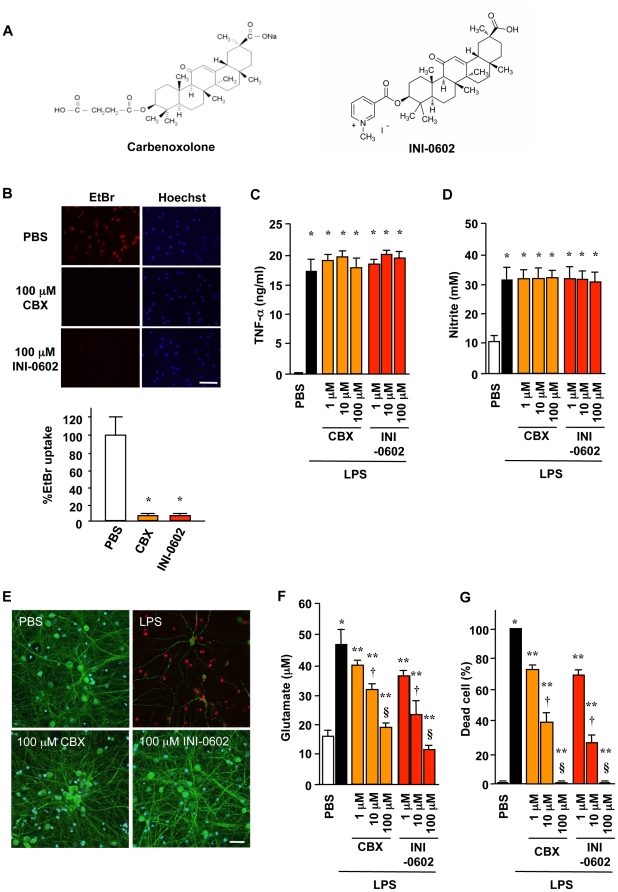
A novel gap junction hemichannel blocker INI-0602 effectively inhibits neurotoxic microglial glutamate release. (A) Structure of carbenoxolone (CBX) and INI-0602. (B) Assessment of microglial hemichannel inhibition by EtBr dye uptake. Scale bar, 100 µm. Data represent the means ± SD (n = 6 per group). *, *P*<0.05 vs. PBS. (C–D) TNF-α production (C) and NO production (D) by microglia treated with 1 µg/ml LPS for 24 h. Data represent the means ± SD (n = 3 per group). *, *P*<0.05 vs. PBS. (E) Fluorescent microscopic images of live/dead staining of mouse primary cortical neuron cultures in microglia-conditioned media. Neurons in microglia-conditioned media containing 1 µg/ml LPS (LPS) underwent cell death with neuritic beading, whereas neurons in microglia-conditioned media containing PBS (PBS), 1 µg/ml LPS plus 100 µM CBX (100 µM CBX), or 1 µg/ml LPS plus 100 µM INI-0602 (100 µM INI-0602) appeared healthy. Green, neuron (MAP2); red, dead cell (PI); blue, nucleus (Hoechst). Scale bar, 20 µm. (F) Glutamate concentrations in media. (G) The percentages of dead neurons. Data represent the means ± SD (n = 6 per group). *, *P*<0.05 vs. PBS; **, *P*<0.05 vs. LPS; †, *P*<0.05 vs. 1 µM; §, *P*<0.05 vs. 10 µM.

**Figure 2:**
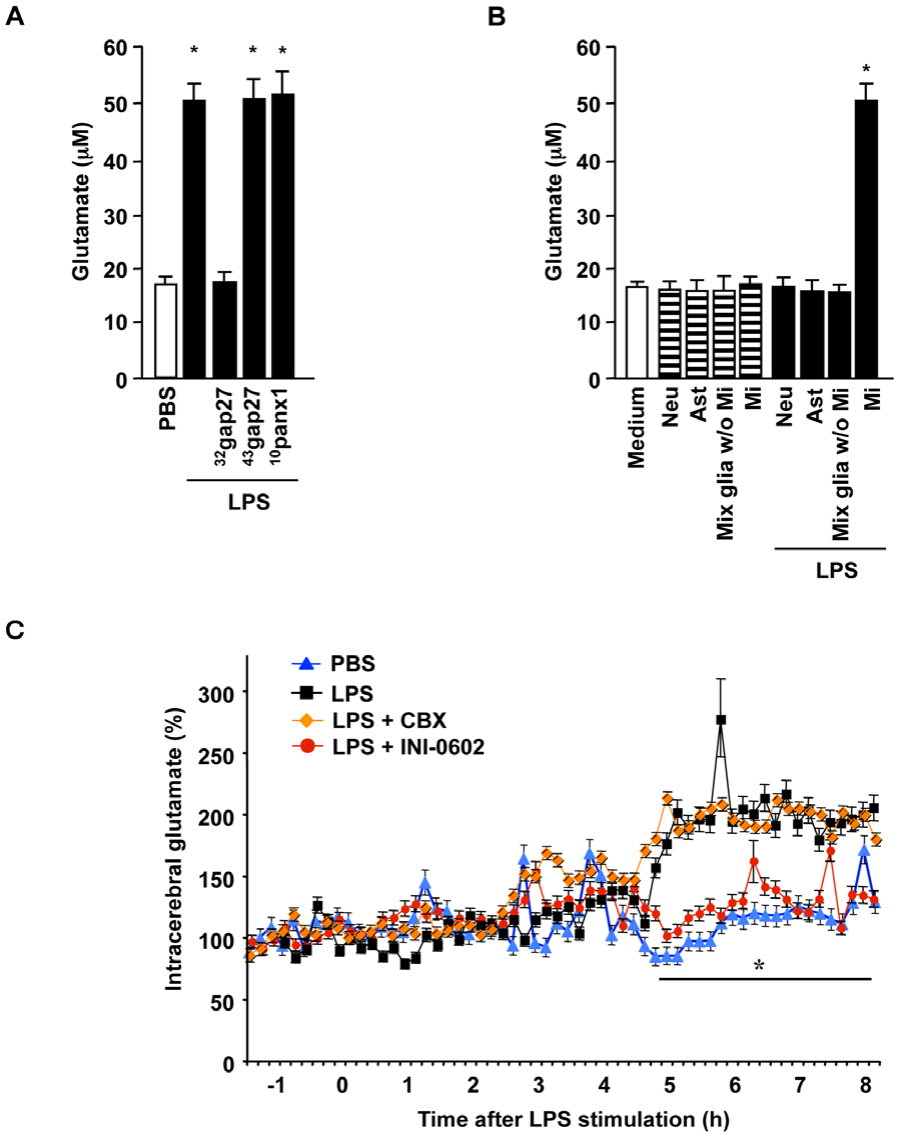
INI-0602 effectively inhibits microglial glutamate release *in vivo*. (A) Glutamate concentrations in microglia conditioned media. Cx32 mimetic peptide reduced microglial glutamate release, which revealed that activated microglia release glutamate from Cx32 hemichannels. Data represent the means ± SD (n = 6 per group). *, *P*<0.05 vs. PBS. (B) Glutamate concentrations in media from primary cell cultures of mouse brain. Only the microglia released a significant amount of glutamate after stimulation with 1 µg/ml LPS for 24 h. Neu, neuron; Ast, astrocyte; Mi, microglia. Data represent the means ± SD (n = 6 per group). *, *P*<0.05 vs. fresh medium. (C) Real-time glutamate concentration monitoring in the hippocampi of mice. All mice were injected LPS at time zero except PBS-treated mice. Blue, PBS-treated mice; black, LPS-treated mice; orange, LPS plus 20 mg/kg CBX-treated mice; red, LPS plus 20 mg/kg INI-0602-treated mice. Data represent the means ± SE (n = 8 per group). *, *P*<0.05 vs. LPS.

Glycyrrhetinic acid derivatives, such as 18α- or 18β-glycyrrhetinic acid and carbenoxolone disodium (CBX), are known to block gap junction hemichannels [25]. These compounds are primary components of licorice extract and are traditionally used as anti-inflammatory drugs. But these glycyrrhetinic acid derivatives hardly penetrate intact BBB. Moreover, long-term treatment with these compounds induces pseudoaldosteronism with hypertension, hypokalemia, and eventual suppression of renin and aldosterone production. To enhance drug penetration into the CNS and circumvent pseudoaldosteronism, we used a previously published method to synthesize various analog drugs of CBX with dihydropyridine conjugates [26]. This approach resulted in a novel gap junction hemichannel blocker, which we named INI-0602 (Figure 1A–B). Although treatment with INI-0602 did not affect morphological change (Figure S1), major cytokine/chemokine expression and NO production (Figure 1C–D and Figure S2) in activated microglia, INI-0602 significantly suppressed lipopolysaccharide (LPS)-induced glutamate release from microglia and subsequent excitotoxic neuronal death in a dose-dependent manner (Figure 1E–G). Since activated microglia release glutamate from Cx32 hemichannels (Figure 2A), INI-0602 was considered to block the hemichannel directly. INI-0602, as well as CBX, was effective to inhibit microglial glutamate release but the apparent toxicity of the compound was much lower than that observed with CBX (Figure S3). We also confirmed that INI-0602 penetrated the CNS more effectively than CBX (Table S1).

We next evaluated the inhibitory effect of INI-0602 on glutamate release *in vivo*. Among the CNS cells, LPS induced glutamate release only from microglia (Figure 2B). INI-0602 significantly ameliorated the elevated glutamate levels in the brains of LPS-treated mice, whereas CBX did not (Figure 2C). These findings agree with the drug distribution data. To identify potential adverse effects of long-term INI-0602 administration, we intraperitoneally injected wild-type C57BL/6J mice with PBS or INI-0602 (5, 10, 20, or 40 mg/kg) every other day for five months. Histological analysis did not detect any obvious differences between the PBS-treated and INI-0602-treated mice (Figure S4). Serologic and urologic examinations also showed no significant abnormalities in these mice (Table S2 and S3). Assessment of 24-h cage activity revealed no significant behavioral changes in these mice (Figure S5). These data suggest that INI-0602 can be used for long-term treatment without precipitating serious adverse effects.

### INI-0602 ameliorates symptoms in two ALS mouse models

**Figure 3:**
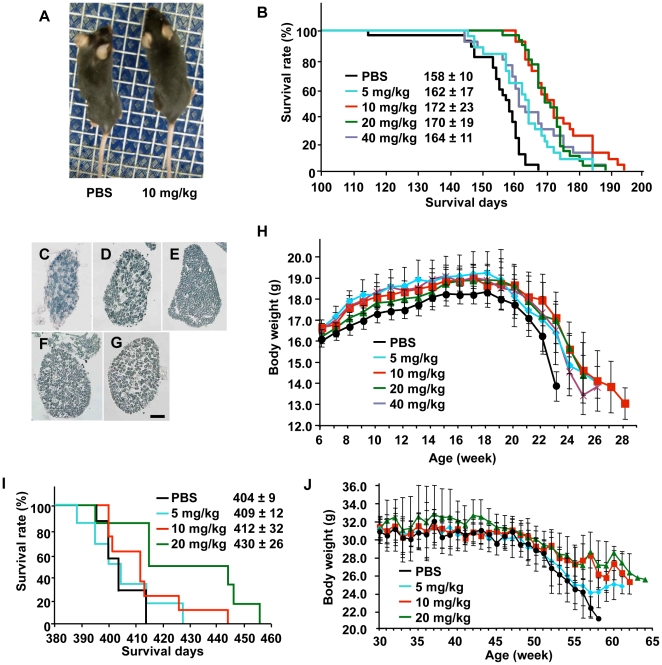
INI-0602 ameliorates disease symptoms in both SOD1 G93A Tg mice and SOD1 G37R Tg mice. (A) A representative photograph of 20-week-old SOD1 G93A Tg mice treated with PBS (left) or 10 mg/kg INI-0602 (right). (B) Survival rates of SOD1 G93A Tg mice (n = 24 per group). Black, PBS; light blue, 5 mg/kg (*P*<0.05); red, 10 mg/kg (*P*<0.00001); green, 20 mg/kg (*P*<0.00001); purple, 40 mg/kg INI-0602 (*P*<0.05). (C–G) Microscopic images of L5 roots from SOD1 G93A Tg mice treated with PBS (C), 5 mg/kg (D), 10 mg/kg (E), 20 mg/kg (F), or 40 mg/kg INI-0602 (G). (H) Body weights of SOD1 G93A Tg mice (n = 24 per group). Black, PBS; light blue, 5 mg/kg (*P*<0.05); red, 10 mg/kg (*P*<0.05); green, 20 mg/kg (*P*<0.05); purple, 40 mg/kg INI-0602 (*P*<0.05). (I) Survival rates and (J) body weights of SOD1 G37R Tg mice (n = 8 per group). Black, PBS; light blue, 5 mg/kg; red, 10 mg/kg (*P*<0.05); green, 20 mg/kg INI-0602 (*P*<0.05).

Next, we administered INI-0602 (5, 10, 20, or 40 mg/kg) to transgenic (Tg) mice carrying a high copy number of a transgene encoding a variant of human superoxide dismutase 1 with a G93A mutation (SOD1 G93A Tg mice), which results in rapid progression of an ALS-like disorder. At 20 weeks old, PBS-treated and INI-0602-treated SOD1 G93A Tg mice showed obvious differences in body size, muscular atrophy, and kyphosis (Figure 3A, Video S1 and S2). Treatment with INI-0602 significantly prolonged the lifespans of the SOD1 G93A Tg mice (5 mg/kg, *P*<0.05; 10 mg/kg, *P*<0.00001; 20 mg/kg, *P*<0.00001; 40 mg/kg, *P*<0.05), whereas all PBS-treated SOD1 G93A Tg mice died by week 25 (Figure 3B). Moreover, INI-0602 treatment prevented axonal loss in the L5 root of the SOD1 G93A Tg mice, whereas PBS-treated SOD1 G93A Tg mice showed markedly fewer axons (Figure 3C–G). PBS-treated SOD1 G93A Tg mice lost significantly more body weight at an earlier age than INI-0602-treated SOD1 G93A Tg mice (*P*<0.05; Figure 3H). Treatment with 10 mg/kg INI-0602 was most effective for all parameters tested.

We also examined the efficacy of INI-0602 in a more slowly progressing model of ALS, in which Tg mice carrying a low copy number of a transgene encoding a G37R mutant variant of human SOD1 (SOD1 G37R Tg mice) [27]. Treatment with INI-0602 significantly prolonged lifespans and prevented body weight loss in SOD1 G37R Tg mice (10 and 20 mg/kg, *P*<0.05; Figure 3I–J).

**Figure 4:**
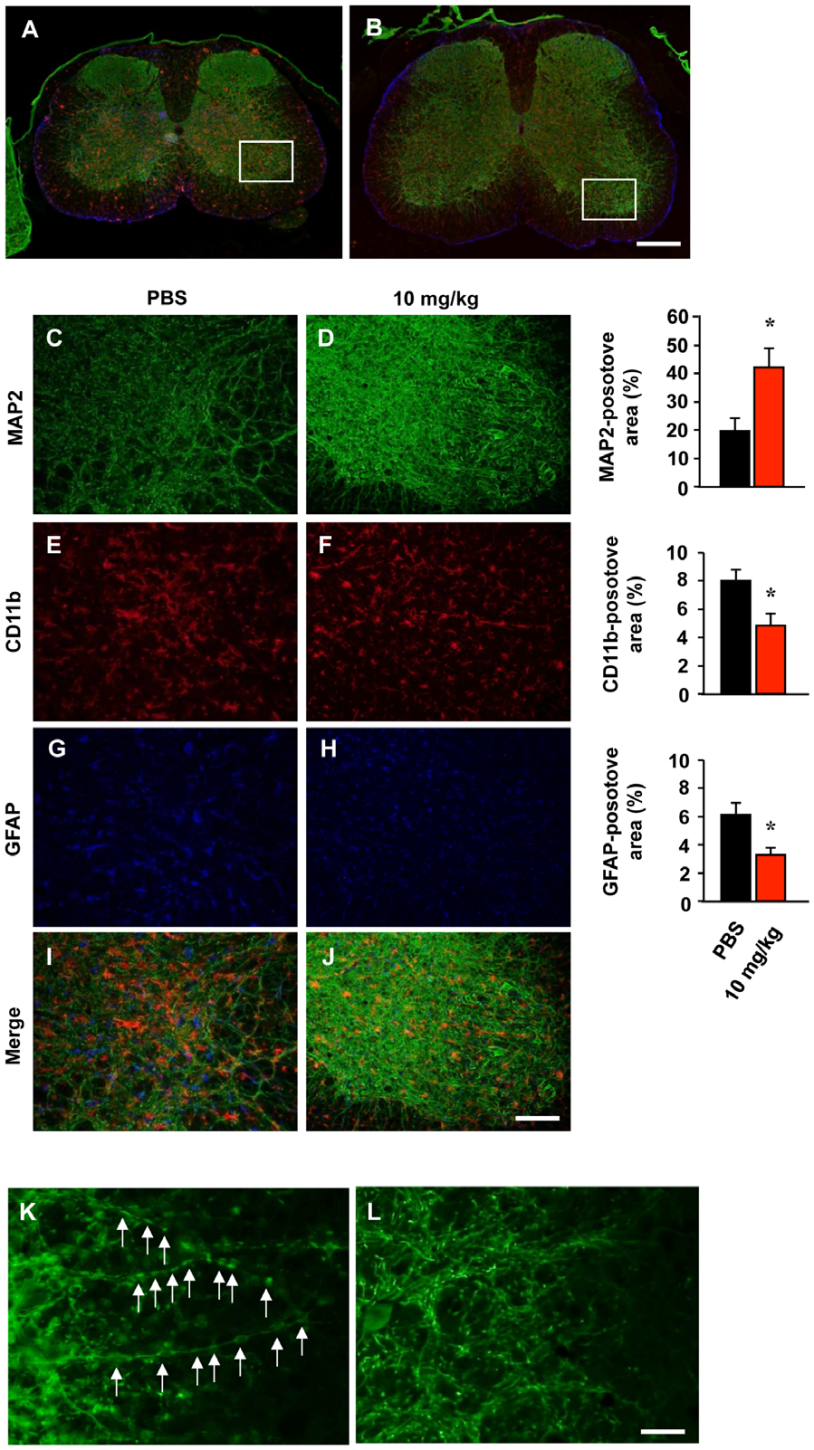
INI-0602 prevents neuronal damage and gliosis in lumber spinal cords of SOD1 G93A Tg mice. (A–B) Fluorescent microscopic images of the lumber spinal cords of 20-week-old SOD1 G93A Tg mice treated with PBS (A) or 10 mg/kg INI-0602 (B). Green, neurons (MAP2); red, microglia (CD11b); blue, astrocytes (GFAP). Scale bar, 200 µm. (C–J) Higher magnification images of the boxed areas in a (C, E, G, and I) and b (D, F, H, and J). Scale bar, 30 µm. Each graph shows the percentage of area occupied by neurons, microglia, and astrocytes, respectively. Data represent the means ± SD (n = 6 per group). *, *P*<0.05 vs. PBS. (K, L) Fluorescent microscopic images of MAP2 staining in the anterior horns of lumber spinal cords from 14-week-old SOD1 G93A Tg mice treated with PBS (K) or 10 mg/kg INI-0602 (L). Arrows indicate neuritic bead formation, an early marker of neuronal dysfunction. Scale bar, 10 µm.

Next, we histologically examined lumber spinal cords from SOD1 G93A Tg mice. Whereas 20-week-old PBS-treated SOD1 G93A Tg mice showed severe atrophy in the anterior horns (Figure 4A) with marked neuronal loss (Figure 4C) and activated microglia (Figure 4E) and astrocytes (Figure 4G), INI-0602 treatment significantly prevented these effects (Figure 4B, D, F, H, J).

Neuritic bead formation is focal swelling in dendrites and axons. This is an early pathologic sign of neuronal damage that precedes neuronal death in a variety of physiologic and pathologic conditions, including ischemia [28], aging [29], and such neurodegenerative diseases as ALS [30], [31] and AD [32]. Neuritic beading reflects impaired dendritic and axonal transport due to intracellular energy loss in neurons [6]. PBS-treated SOD1 G93A Tg mice at 14 weeks old (early–moderate phase of disease) exhibited profound neuritic beading in the anterior horns of their spinal cords (Figure 4K, arrows), which was morphologically similar to spheroid structures observed in ALS patients [30], [31]. Interestingly, INI-0602 treatment markedly reduced this neuritic beading in the anterior horns (Figure 4L).

### INI-0602 alleviates symptoms in a mouse model of AD

**Figure 5:**
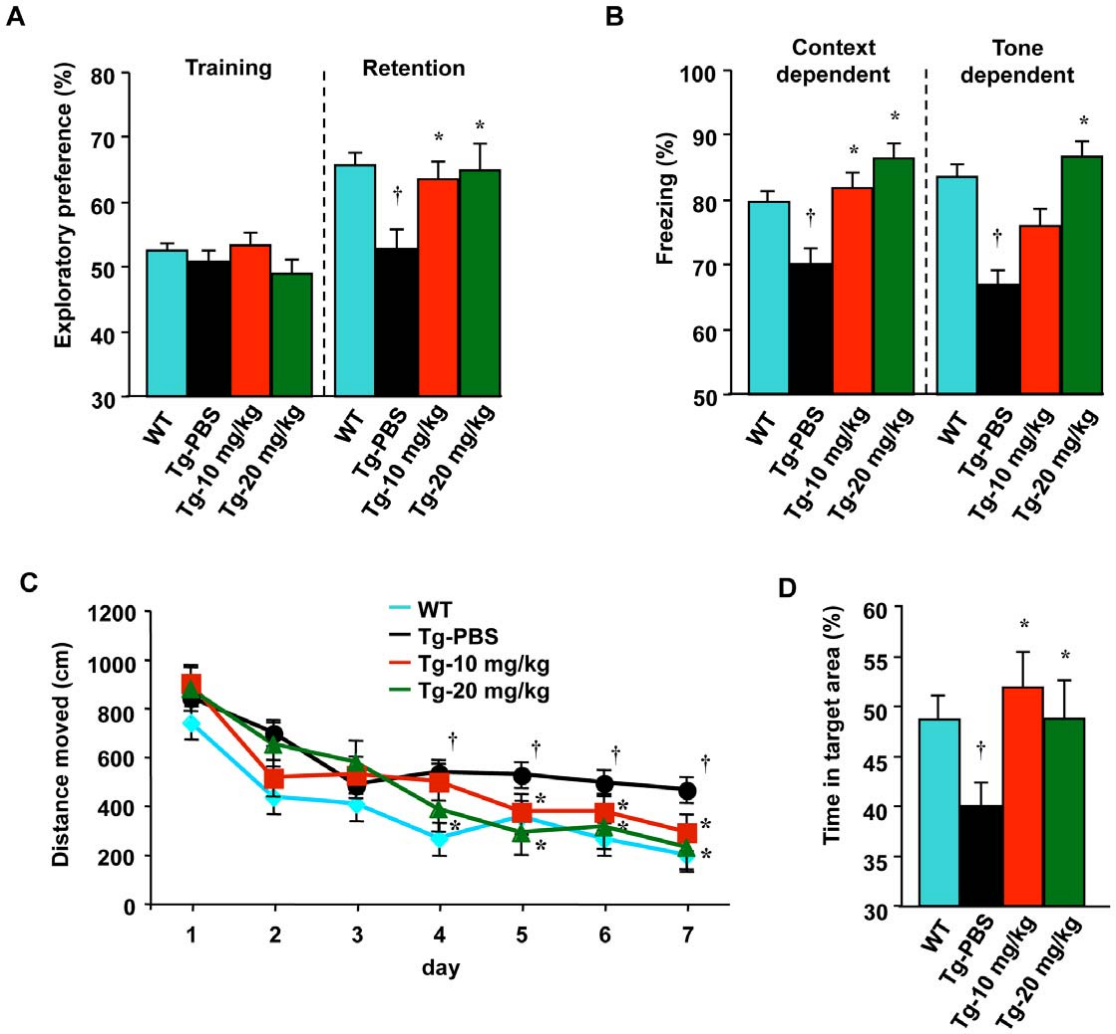
INI-0602 improves memory deficits in APP/PS1 Tg mice. (A) Object recognition memory assessment by a novel-object recognition test using 9-month-old mice. Data represent the means ± SD (WT, wild-type mice, n = 22; Tg-PBS, PBS-treated Tg mice, n = 22; Tg-10 mg/kg, 10 mg/kg INI-0602-treated Tg mice, n = 16; Tg-20 mg/kg, 20 mg/kg INI-0602-treated Tg mice, n = 15). *, *P*<0.05 vs. WT; †, *P*<0.05 vs. Tg-PBS. (B) Associative learning assessment by cued and contextual fear conditioning tests using 10-month-old mice. We assessed the same cohort of mice used in A. Data represent the means ± SD (WT, n = 13; Tg-PBS, n = 13; Tg-10 mg/kg, n = 16; Tg-20 mg/kg, n = 15). *, *P*<0.05 vs. WT; †, *P*<0.05 vs. Tg-PBS. (C–D) Reference memory assessment using the distance moved (C) and the percentage of time spent searching (D) during a 60-sec session in a Morris water maze test using 11-month-old mice. We assessed the same cohort of mice used in A. Data represent the means ± SD (WT, n = 22; Tg-PBS, n = 22; Tg-10 mg/kg, n = 16; Tg-20 mg/kg, n = 15). *, *P*<0.05 vs. WT; †, *P*<0.05 vs. Tg-PBS.

We also examined whether blocking microglial glutamate release improved cognitive dysfunction in an AD mouse model using transgenic mice expressing mutant variants of human amyloid precursor protein (APP) and presenilin 1 (PS1) (APP/PS1 Tg mice). First, we investigated recognition memory in 9-month-old mice using a novel-object recognition test. Compared with wild-type mice, PBS-treated APP/PS1 Tg mice displayed reduced exploratory preference for the novel object in the retention session (*P*<0.05; Figure 5A), an indication of impaired recognition memory. In contrast, INI-0602 treatment (10 or 20 mg/kg) significantly reversed this impairment in the APP/PS1 Tg mice (*P*<0.05; Figure 5A). Next, we evaluated associative learning in 10-month-old mice using a conditioned fear learning test. During the preconditioning phase (training), the mice rarely responded by freezing. There were no differences among the groups in the basal frequency of freezing responses (data not shown). Wild-type mice showed a marked contextual freezing response 24 h after fear conditioning (Figure 5B). PBS-treated APP/PS1 Tg mice, on the other hand, exhibited less freezing in the contextual tests (*P*<0.05; Figure 5B), which suggested impaired associative learning. APP/PS1 Tg mice treated with INI-0602 (10 or 20 mg/kg) were indistinguishable from wild-type mice in these tasks, and INI-0602 treatment significantly prevented the contextual freezing phenotype observed in PBS-treated APP/PS1 Tg mice (*P*<0.05; Figure 5B). In a cued (tone) learning test, PBS-treated APP/PS1 Tg mice also froze less 24 h after fear conditioning compared with wild-type mice (*P*<0.05; Figure 5B). INI-0602 treatment (20 mg/kg) significantly prevented the reduced cued freezing phenotype (*P*<0.05; Figure 5B). We also assessed reference memory in 11-month-old mice using a Morris water maze test. Reference memory appeared to be impaired in PBS-treated APP/PS1 Tg mice, which took significantly longer and traveled significantly farther to reach the hidden platform (*P*<0.05 compared with wild-type mice; Figure 5, C and D). INI-0602-treated APP/PS1 Tg mice (10 or 20 mg/kg) were indistinguishable from wild-type mice in this test, suggesting INI-0602 treatment significantly alleviated the reference memory deficits (*P*<0.05; Figure 5, C and D). No alterations in nociceptive responses were observed in any APP/PS1 Tg mice: the minimal current required to elicit flinching/running, jumping, or vocalizing was the same for the various mice (data not shown).

**Figure 6:**
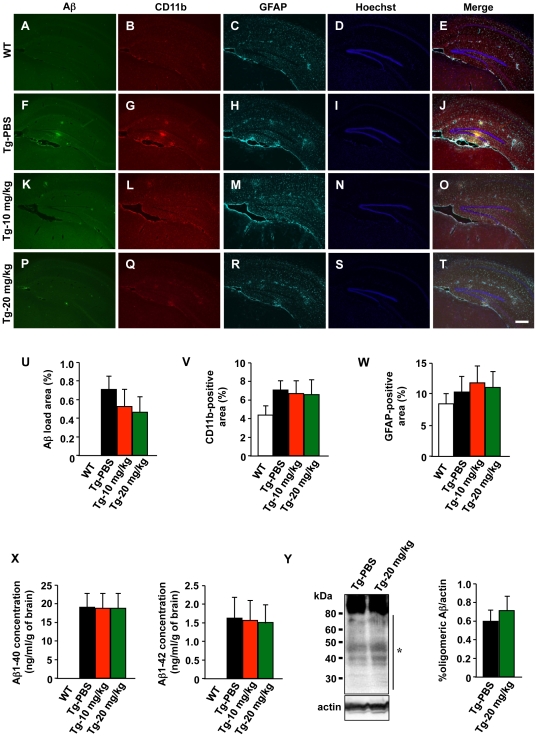
INI-0602 does not affect Aβ deposition or glial activation. (A–T) Fluorescent microscopic images of hippocampi from 11-month-old APP/PS1 Tg mice. Scale bar, 200 µm. (U–W) Percentage of area occupied by Aß (U), microglia (V), and astrocytes (W). Data represent the means ± SD (n = 6 per group). (X) Human Aß1–40- and Aß1–42-specific ELISAs using homogenized brains from 11-month-old APP/PS1 Tg mice. Data represent the means ± SD (n = 6 per group). (Y) Western blot analysis of oligomeric Aβ extracted from 11-month-old APP/PS1 Tg mice. *, oligomeric Aβ. Data represent the means ± SD (n = 3 per group). WT, wild-type mice; Tg-PBS, PBS-treated Tg mice; Tg-10 mg/kg, 10 mg/kg INI-0602-treated Tg mice; Tg-20 mg/kg, 20 mg/kg INI-0602-treated Tg mice.

To examine whether blocking microglial glutamate release affected amyloid β (Aβ) deposition, we evaluated Aβ load in the brains of 11-month-old APP/PS1 Tg mice using immunostaining and ELISAs. Whereas no Aβ deposition or glial activation was observed in wild-type mice (Figure 6, A–C and U), PBS-treated APP/PS1 Tg mice had Aβ deposits surrounded by activated microglia and astrocytes (Figure 6F–H, U). PBS-treated APP/PS1 Tg mice showed no obvious neuronal loss in the hippocampus (Figure 6I), which agreed with previous reports [33], [34]. Despite a significantly reduction in cognitive impairments, INI-0602 treatment did not significantly alter Aβ deposition or glial activation (Figure 6K–M, P–R, U–W). Human Aβ specific ELISAs also revealed no difference in the Aβ load among PBS-treated and INI-0602-treated APP/PS1 Tg mice (Figure 6X). Western blot analysis also showed no difference in the amount of soluble oligomeric Aβ1–42 (Figure 6Y), which has been reported to exhibit higher neurotoxicity than fibrillar Aβ [35]–[37]. These findings agreed with *in vitro* observations showing that hemichannel blockade neither enhanced microglial Aβ uptake nor altered microglial activation (data not shown).

## Discussion

In the present study, we synthesized a novel gap junction hemichannel blocker INI-0602 based on CBX using dihydropyridine conjugates as a chemical drug delivery system to enhance BBB penetration. In general, dihydropyridine conjugates serve the drug molecule with the sufficient lipophilicity to enter the CNS. Then, dihydropyridine conjugates undergo chemical conversion to pyridinium salt by *in vivo* redox system. This conversion promotes retention of the drug molecule in the CNS and it is also expected that this conversion accelerate peripheral elimination of the drug molecule distribution outside the CNS. Thus, dihydropyridine conjugates may not only lead to effective drug delivery into the CNS but also decrease the drug toxicity due to the effect in the periphery [26]. In fact, we confirmed here that dihydropyridine conjugates gave INI-0602 the advantage of BBB penetration, CNS retention and low toxicity over CBX. Unexpectedly, INI-0602 has a short half life *in vivo* (9 min in the blood and 25 min in the CNS, respectively) although INI-0602 showed a strong effect *in vivo* even by every other day treatment. We confirmed that only 30-min treatment with INI-0602, but not CBX, significantly suppressed LPS-stimulated microglial glutamate release in the following 48 h *in vitro* (H. Takeuchi, unpublished data). It suggested that INI-0602 induced long-term inactivation or internalization of gap junction/hemichannel. However, further studies are needed to elucidate this issue.

Like other cell types, microglia maintain a resting physiologic glutamate level via the glutamate dehydrogenase pathway without significant secretion of glutamate [38]–[41]. In the activated state, microglia produce a large amount of glutamate through an upregulation of glutaminase expression, rather than glutamate dehydrogenase. The activated microglia then secrete high levels of glutamate through Cx32 hemichannels [5], [21]–[24]. Interestingly, inflammatory cytokines, especially tumor necrosis factor-α and interferon-γ, enhance microglial glutaminase expression, glutamate production, and cell-surface expression of gap junction hemichannels [5], [42]. Synergistic and autocrine activities of these molecules may cause the release of large amounts of glutamate to induce excitotoxic neuronal death. Activated microglia undergo dramatic morphologic changes and movements. Accordingly, hemichannels in activated microglia may be more exposed to the extracellular space and provide a unique surface for molecular drug target than those in unactivated microglia and other CNS cells. Thus, an inhibitor of hemichannel may specifically suppress glutamate release from microglia, and this specificity may translate into fewer adverse effects during treatment. Moreover, neuronal and astrocytic gap junctions are also thought to contribute to the spread of neuronal damage in neurological disorder models such as brain ischemia, migraine, and epilepsy [43]–[48]. Therefore blockade of neuronal and astrocytic gap junction/hemichannel may elicit additive neuroprotective effects. Recent reports demonstrated that activated astrocytes also play an important role in neuronal death by releasing soluble factors in ALS models *in vitro* and *in vivo* [49]–[51]. Another recent study reported that Aβ dramatically enhanced gap junction/hemichannel-mediated Ca^2+^ wave propagation in astrocytes that may contribute to AD pathology [52]. Thus blockade of gap junction in astrocytes besides microglia may contribute to enhance neuroprotective effect. On the other hand, excessive inhibition of gap junction/hemichannel may induce dysfunction of neural system because gap junction/hemichannel also contribute to maintaining homeostasis in neuron-glia interaction [53]. We are planning more thorough studies to investigate these issues.

In a murine model of ALS, degenerating neurons are thought to secrete unidentified factor(s) and activate microglia and astrocytes, thereby exacerbating neurodegeneration [27], [51]. Microglial activation may also be upstream of this vicious feed-forward cycle of neurodegeneration and glial activation [1], which is likely fueled by microglia-derived glutamate. In this study, INI-0602 treatment suppressed both neuronal loss and glial activation in an ALS model while INI-0602 itself did not alter microglial activation. Thus we suggest that INI-0602 halted this feed-forward cycle and reduced glial activation indirectly by inhibiting microglial glutamate release. In AD model mice, no obvious neuronal loss was observed [33], suggesting that inhibition of glutamate release from microglia may improve neuronal dysfunction in the hippocampus via reduced NMDA-mediated neurotoxicity. Other NMDA receptor antagonists also have similar effects in AD patients [12]. It is still possible, however, that the vicious feed-forward cycle described above may contribute to AD pathology because AD patients exhibit severe neuronal loss and glial activation [1]. Additionally, INI-0602 treatment did not significantly alter Aβ load despite cognitive improvement. In recent animal models and human clinical trials, cognitive improvements in response to treatments were minimally related to changes in Aβ load [54]–[56]. Our findings also suggest that factors through gap junction hemichannels (including microglial glutamate) in addition to Aβ neuropathology may be important determinants of cognitive decline in AD patients.

In conclusion, we have generated a CNS penetratable gap junction hemichannel blocker and have demonstrated that pharmacologic blockade of gap junction hemichannels effectively attenuated glutamate release from neurotoxic activated microglia and ameliorated symptoms of mouse models of ALS and AD. Thus, gap junction hemichannel blockers may provide a new therapeutic approach to prevent microglia-mediated, non-cell autonomous neuronal death in neurodegenerative diseases including ALS and AD.

## Methods

### Reagents

All reagents were obtained from Sigma-Aldrich unless specified otherwise. The gap junction hemichannel blocker INI-0602 (IUPAC name: 3-(((3S,4aR,6aR,6bS,8aS,11S,12aR,14aR,14bS)-11-carboxy-4,4,6a,6b,8a,11,14b-heptamethyl-14-oxo-1,2,3,4,4a,5,6,6a,6b,7,8,8a,9,10,11,12,12a,14,14a,14b-icosahydropicen-3-yloxy) carbonyl)-1-methylpyridinium iodide) was developed by H. Takeuchi and A. Suzumura (Patent: WO/2010/007788). In brief, nicotinoyl chloride hydro chloride salt was reacted with 18β-glycyrrhetinic acid, thereby introducing nicotinate to the hydroxyl group of the 18β-glycyrrhetinic acid followed by attaching an alkyl group to the nitrogen atom of the pyridine ring with methyl iodide. Then, INI-0602 was isolated as a pyridinium salt. INI-0602 used in this study was chemically synthesized and purified by the Nard Institute (Osaka, Japan). The purity of the compound was more than 99%.

### Animals

This study was carried out in strict accordance with the guideline for the care and use of laboratory animals of Nagoya University. All protocols for animal experiments were approved by the Animal Experiment Committee of Nagoya University (permit number: 651 and 652). Transgenic mice expressing mutant variants of human amyloid precursor protein (APP) with K595N and M596L mutations and presenilin 1 (PS1) with A264E mutation [33] were purchased from the Jackson Laboratory (B6C3-Tg(APP695)3Dbo Tg(PSEN1)5Dbo/J; #003378) and were backcrossed to C57BL/6J mice for more than 10 generations after purchase (here designated as APP/PS1 Tg mice). Transgenic mice carrying a high copy number of a transgene encoding a G93A mutant of human superoxide dismutase 1 (SOD1) [57] (here designated as SOD1 G93A Tg mice) were also purchased from the Jackson Laboratory (B6.Cg-Tg(SOD1-G93A)1Gur/J; #004435). Transgenic mice carrying a low copy number of a transgene encoding a G37R mutant of human SOD1 [27] (here designated as SOD1 G37R Tg mice) were provided by Dr. D. W. Cleveland (University of California, San Diego, USA).

### Cells

Mouse primary cultures of cortical neurons were prepared from C57BL/6J mice as described previously [5], [6]. Cortices were dissected and freed of meninges. Cortical fragments were dissociated into single cells using dissociation solution, and they were resuspended in Neuron Medium (serum-free conditioned medium from 48-h rat astrocyte confluent cultures based on Dulbecco's modified Eagle's minimum essential medium (DMEM)/F12 with N2 supplement, Sumitomo Bakelite, Akita, Japan). Primary neuronal cells were plated on 12 mm-poly-ethyleneimine-coated coverslips (Asahi Techno Glass Corporation, Chiba, Japan). The purity of the cultures was more than 95% as determined by NeuN-specific immunostaining. Mouse primary microglia were isolated from primary mixed glial cell cultures from newborn C57BL/6J mice on the 14th day with the ‘shaking off’ method as described previously [58]. The purity of the cultures was 97 to 100% as determined by CD11b-specific immunostaining. Cultures were maintained in DMEM supplemented with 10% fetal bovine serum (JRH Biosciences, Lenexa, KS, USA), 5 µg/ml bovine insulin, and 0.2% glucose. Mouse primary astrocytes were also prepared from primary mixed glial cell cultures from newborn C57BL/6J mice on the 14th day by three to four repetitions of trypsinization and replating as described previously [21]. The purity of the cultures was more than 95% as determined by glial fibrillary acidic protein (GFAP)-specific immunostaining. Astrocyte cultures were maintained with same medium as microglia cultures. Cells were plated at a density of 5×10^4^ cells per well in 24-well multidishes. For stimulation, cells were incubated with 1 µg/ml LPS for 24 h. To assess neuroprotective effects, microglia were cultured in Neuron Medium containing 1 µg/ml LPS with each drug as follows: 1–100 µM CBX; 1–100 µM INI-0602; connexin (Cx) 32 mimetic peptide, 0.25 mg/l ^32^gap 27 (SRPTEKTVFT, Thermo Electron GmbH); Cx43 mimetic peptide, 0.25 mg/l ^43^gap 27 (SRPTEKTIFII, Thermo Electron GmbH); pannexin 1 mimetic peptide, 0.25 mg/l ^10^panx1 (WRQAAFVDSY, Thermo Electron GmbH). To assess IC_50_ of drugs, microglia were cultured in Neuron Medium containing 1 µg/ml LPS with 0.1–500 µM CBX or INI-0602. After a 24-h incubation, we assessed the glutamate concentration in microglia-conditioned medium and applied it to each well containing neurons after 10–13 days *in vitro*. Neurotoxicity was evaluated 24 h after application.

### Immunocytochemistry

Neuron cultures were live-stained with 0.1 µg/ml Hoechst 33342 and 2 µg/ml propidium iodide (PI) as described previously [6]. After rinsing with Neuron Medium three times, cells were fixed in 4% paraformaldehyde for 30 min, permeabilized in 0.05% Triton X-100 for 10 min, and stained using mouse monoclonal anti-microtubule-associated protein 2 (MAP2) antibody (MAB364, Chemicon, Temecula, CA, USA) and Alexa 488–conjugated secondary antibodies (Molecular Probes, Eugene, OR, USA). More than 100 neurons in duplicate wells were assessed blindly in six independent trials with a deconvolution fluorescence microscope system (Keyence, Osaka, Japan). The ratio of dead cells was calculated as a percentage of PI-positive cells among Hoechst-positive cells.

### Assessment of microglial hemichannel inhibition

To assess microglial hemichannel inhibition, we used ethidium bromide (EtBr) dye uptake method as described previously [59], [60]. Briefly, mouse primary microglia were plated at a density of 1×10^5^ cells per well in 24-well multidishes. They were pretreated with DMEM containing 100 µM CBX or INI-0602 for 30 min. Then 10 µM EtBr was added. After 1 h incubation, cells were gently rinsed with phosphate buffered saline (PBS) three times and fixed with fixed in 4% paraformaldehyde for 30 min. Cells were counterstained with 0.1 µg/ml Hoechst 33342. EtBr fluorescence of more than 100 cells was analyzed with a deconvolution fluorescence microscope system (Keyence, Osaka, Japan) in six independent trials. Relative dye uptake was calculated as EtBr fluorescence divided by Hoechst-positive cell number. The ratio of dye uptake was calculated as a percentage of relative dye uptake in PBS-treated cells.

### Assessment of glutamate release

To measure extracellular glutamate concentrations *in vitro*, we used a colorimetric Glutamate Assay Kit (Yamasa Corporation, Tokyo, Japan) as described previously [6].

### Assessment of tumor necrosis factor-α (TNF-α) production

To measure microglial TNF-α production, we used a mouse TNF-α specific ELISA kit (BD Pharmingen, Franklin Lakes, NJ, USA) as described previously [61].

### Assessment of NO production

To measure microglial NO production, we performed the Griess reaction on the media as described previously [6].

### RNA extraction and reverse transcription (RT)-PCR

To examine microglial mRNA expression of TNF-α, interleukin (IL)-1β, IL-6, CCL2, CCL5, CXCL10 and glyceraldehyde-3-phosphate dehydrogenase (GAPDH, as an internal control), we performed RNA extraction and RT-PCR analysis with each specific primer as described previously [22].

### Assessment of drug concentration

To assess the drug concentrations from plasma and brain tissues, mice were intravenously injected with 20 mg/kg CBX or INI-0602. Plasma and brain tissues were collected at the indicated time points after injection. Drug concentrations were assessed using a high-performance liquid chromatography (HPLC)/mass spectrometry (MS)/MS system (Applied Biosystems Japan, Tokyo, Japan).

### Drug administration

To examine acute drug toxicity, 12-week-old C57BL/6J mice were given single intraperitoneal injection with PBS, CBX or INI-0602 at the indicated doses. To assess chronic drug toxicity, 12-week-old C57BL/6J mice were intraperitoneally treated with 20 mg/kg CBX or INI-0602 every other day for five months. To evaluate drug efficacy, mice were intraperitoneally injected with the indicated doses of INI-0602 every other day. SOD G93A Tg mice were treated beginning the week after the onset of hindlimb motor deficits (approximately 8 weeks old), which is an early symptom in SOD1 G93A Tg mice [62]. SOD G37R Tg mice were treated beginning the week after the onset of body weight loss (approximately 7 months old), which is an early symptom in SOD1 G37R Tg mice [51]. APP/PS1 Tg mice were treated beginning the week after the onset of Aβ deposition in the brain (approximately 4 months old).

### *In vivo* microdialysis

We performed real-time monitoring of glutamate or acetylcholine concentrations in the hippocampi of mice using *in vivo* microdialysis as described previously [63]. To stimulate glutamate release, 10 µg of LPS in 2 µl of artificial CSF (aCSF: 147 mM NaCl, 4 mM KCl, and 2.3 mM CaCl_2_) were injected through the microinjection tube at a flow rate of 1.0 µl/min. Mice were administered with 20 mg/kg CBX or INI-0602 intraperitoneally 30 min before stimulation. Levels of glutamate in the dialysates were measured with a HPLC system (Eicom, Kyoto, Japan). We used the means of the measured levels for 90 min before LPS stimulation as baseline level of each group. The results are expressed as the percentage of the measured levels to baseline level.

### Behavioral tests

We measured cage activity of mice for 24 h using an infrared ray sensor monitor system (Neuroscience, Yokohama, Japan) as described previously [64]. To assess memory impairments, 9- to 11-month-old APP/PS1 Tg mice treated with PBS or INI-0602 were examined in a novel-object recognition test, cued and contextual fear conditioning tests, and a Morris water maze test as described previously [34], [65]. The novel-object recognition test procedure consisted of three sessions: habituation, training, and retention. Each mouse was individually habituated to the box (a black Plexiglas box with abundant wood chips, 30×30×40 high cm) with 10 min of exploration in the absence of objects for 3 consecutive days (habituation session, days 1–3). During the training session, two novel objects were symmetrically fixed to the floor of the box, 8 cm from the walls, and each animal was allowed to explore in the box for 10 min (day 5). The objects were constructed from a golf ball, wooden column, and wooden triangular pyramid. They were different in shape and color but similar in size. An animal was considered to be exploring the object when its head was facing the object or it was touching or sniffing the object. The time spent exploring each object was recorded. After training, mice were immediately returned to their home cages. During the retention sessions (day 6), the animals were placed back into the same box 24 h after the training session, but one of the familiar objects used during training had been replaced with a novel object. The animals were then allowed to explore freely for 5 min and the time spent exploring each object was recorded. Throughout the experiments, the objects were used in a counterbalanced manner. A preference index in the retention session, a ratio of the amount of time spent exploring the novel object over the total time spent exploring both objects, was used to measure cognitive function. In the training session, the preference index was calculated as a ratio of the time spent exploring the object that was replaced by the novel object in the retention session, over the total time exploring. In the cued and contextual fear conditioning tests, to measure basal levels of freezing response (preconditioning phase), mice were individually placed in a neutral cage (a black Plexiglas box with abundant wood chips, 30×30×40 high cm) for 1 min, then in the conditioning cage (a transparent Plexiglas box, 30×30×40 high cm) for 2 min. For training (conditioning phase), mice were placed in the conditioning cage, then a 15 s tone (80 dB) was delivered as a conditioned stimulus. During the last 5 s of the tone stimulus, a foot shock of 0.6 mA was delivered as an unconditioned stimulus through a shock generator (Brain Science Idea, Osaka, Japan). This procedure was repeated four times with 15 s intervals. Cued and contextual tests were carried out 1 day after fear conditioning. For the contextual test, mice were placed in the conditioning cage and the freezing response was measured for 2 min in the absence of the conditioned stimulus. For the cued test, the freezing response was measured in the neutral cage for 1 min in the presence of a continuous-tone stimulus identical to the conditioned stimulus. The Morris water maze test was conducted in a circular pool 1.2 m in diameter and filled with water at 22±1°C. A hidden platform (7 cm in diameter) was used. The mice were given three trials (one block) for 7 consecutive days, during which the platform was left in the same position. The time taken to locate the escape platform (escape latency) and the distance moved was determined in each trial by using the SMART system (Panlab, Barcelona, Spain). One day after the last training trial, the mice were given a probe test without the platform and allowed 60 s to search the pool. We separated the pool in four quadrants (target (platform), left, opposite, and right) and assessed the time spent to search in each quadrant.

### Histological analysis

Ten-micrometer-thick frozen sections of mouse organs were prepared using a previously described method [34]. Sections of organs except brain and spinal cord were stained with hematoxylin and eosin using standard procedures. Brain and spinal cord sections were permeabilized with 1% Triton X-100 after blocking with 10% normal goat serum for 30 min, and they were stained with specific primary antibodies for neurons (mouse monoclonal anti-MAP2 antibody, MAB364, Chemicon), microglia (rat monoclonal anti-CD11b antibody, clone M1/70.15, AbD Serotec, Raleigh, NC, USA), astrocytes (rabbit polyclonal anti-glial fibrillary acidic protein (GFAP) antibody, Dako, Glostrup, Denmark), and amyloid β (Aβ) (mouse monoclonal antibody, clone 4G8, Chemicon). L5 root sections were stained with 0.5% Sudan black for myelin stain. Images were analyzed with a deconvolution fluorescence microscope system (Keyence) as reported previously [34].

### Human Aβ ELISA

To evaluate the amount of human Aβ1–40 and Aβ1–42 in mouse brains, we used a human Aβ1–40 and Aβ1–42 specific ELISA kit (Wako Pure Chemical Industries, Osaka, Japan) according to the manufacturer's protocol as described previously [65]. Brains were homogenized with 70% formic acid and centrifuged at 100,000 *g* for 1 h. The supernatants of insoluble 70% formic acid extracts were neutralized with 1 M Tris-HCl, pH 8.0 at a dilution of 1∶20 and were measured by each Aβ specific ELISA kit. The values obtained were corrected with the wet weight of each brain sample.

### Western blotting

Oligomeric Aβ in APP/PS1 Tg mouse brain was extracted from the soluble, extracellular-enriched fraction as described previously[34]. Proteins were separated on a 5–20% Tris-glycine SDS-polyacrylamide gel and transferred to Hybond-P PVDF membrane (GE Healthcare UK Ltd. Buckinghamshire, UK). Blots were incubated in mouse anti-Aβ monoclonal antibody (clone 6E10, 1∶1000, Chemicon) overnight at 4°C. Bound antibody was visualized using horseradish peroxidase-conjugated anti-mouse IgG (1∶5000, GE Healthcare) and SuperSignal West Pico Chemiluminescent Substrate (Thermo Fisher Scientific, Rockford, IL). The intensity of the bands was calculated by using CS Analyzer 1.0 (Atto Corporation, Tokyo, Japan).

### Statistics and analysis

Biochemical and behavioral data were statistically analyzed using one-way ANOVA followed by post hoc Tukey tests. The survival rate was assessed using Kaplan-Meier and log-rank tests. All results were analyzed using GraphPad Prism (GraphPad Software).

## Supporting Information

Figure S1 **Phase contrast images of microglia.** (A) Activated microglia treated with 1 µg/ml LPS for 24 h. (B) Activated microglia treated with 1 µg/ml LPS and 100 µM INI-0602 for 24 h. No morphological difference was observed between both activated microglia. Scale bar; 20 µm.
(TIF)

Figure S2 **Major cytokine/chemokine expression by microglia treated with gap junction hemichannel blockers.** Representative RT-PCR data of major cytokine/chemokine in microglia. Microglia were treated 1 µg/ml LPS for stimulation. To assess drug effects, cells were simultaneously treated with 1–100 µM CBX or INI-0602. Assessments were performed 24 h after treatment.
(TIF)

Figure S3 **IC_50_ and LD_50_ of INI-0602.** (A) Inhibition-concentration curves of CBX and INI-0602 for microglial glutamate release. Data represent the means ± SD (n = 6 per group). (B) IC_50_ *in vitro* (n = 6 per group) and LD_50_ *in vivo* (n = 10 per group) of CBX and INI-0602.
(TIF)

Figure S4 **Hematoxylin and eosin staining images of major organs from mice treated with PBS or INI-0602.** Representative hematoxylin and eosin staining images of the heart, kidney liver and spleen from 12-week-old C57BL/6J mice treated with PBS or 20 mg/kg INI-0602 every other day for five months. No significant differences were observed. Scale bar, 200 µm.
(TIF)

Figure S5 **Assessment of 24-h cage activity of mice treated with PBS or INI-0602.** Representative 24-h cage activity data of 12-week-old C57BL/6J mice treated with PBS or 20 mg/kg INI-0602 every other day for five months. No significant difference was observed. Data represent the means ± SD (n = 8 per group).
(TIF)

Table S1 **Drug concentrations from plasma and brain tissues.** Mice were intravenously injected with 20 mg/kg CBX or INI-0602. Drug concentrations were assessed using HPLC/MS. ND, not detected.
(DOC)

Table S2 **Blood analysis of 12-week-old mice treated with PBS or INI-0602.** Wild-type C57BL6/J mice were treated with PBS or INI-0602 (5, 10, 20, or 40 mg/kg) every other day for five months. Blood was collected from the inferior aorta under deep anesthesia. Whole blood and serum were analyzed for the items as below with an autoanalyzer (Nagahama Life Science Laboratory, Nagahama, Japan). Data represent the means ± SE (n = 10 per group).
(DOC)

Table S3 **Urinalysis of 12-week-old mice treated with PBS or INI-0602.** Wild-type C57BL6/J mice were treated with PBS or INI-0602 (5, 10, 20, or 40 mg/kg) every other day for five months. Urine was collected from the bladder under deep anesthesia and was immediately assessed using Uropaper III (Eiken Chemical, Tokyo, Japan). Data represent the means ± SE (n = 10 per group). ND, not detected.
(DOC)

Video S1 **A representative movie of a 20-week-old SOD1 G93A Tg mouse treated with PBS.** The mouse shows severe muscular atrophy and kyphosis, and is hardly able to walk.
(MOV)

Video S2 **A representative movie of a 20-week-old SOD1 G93A Tg mouse treated with 10 mg/kg INI-0602.** The mouse shows motor weakness but is still able to walk steadily.
(MOV)
