# Supplementary figures and images for: Blockade of Gap Junction Hemichannel Suppresses Disease Progression in Mouse Models of Amyotrophic Lateral Sclerosis and Alzheimer's Disease
Source: PLoS One. 2011 Jun 21;6(6):e21108. doi: 10.1371/journal.pone.0021108 (PMC3119678; doi:10.1371/journal.pone.0021108)

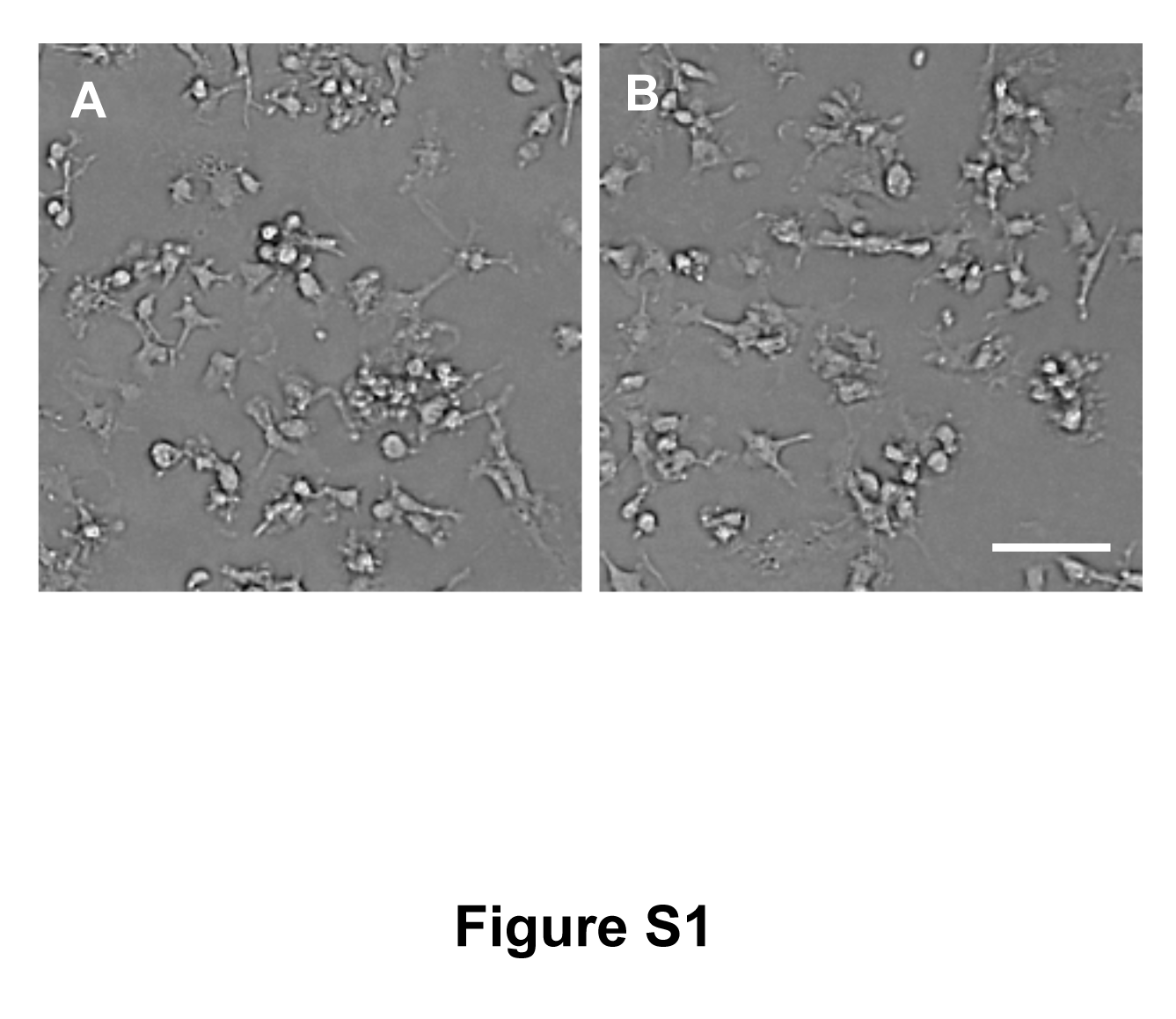

Supplement: Figure S1 — Phase contrast images of microglia. (A) Activated microglia treated with 1 µg/ml LPS for 24 h. (B) Activated microglia treated with 1 µg/ml LPS and 100 µM INI-0602 for 24 h. No morphological difference was observed between both activated microglia. Scale bar; 20 µm. (TIF) [file pone.0021108.s001.tif]

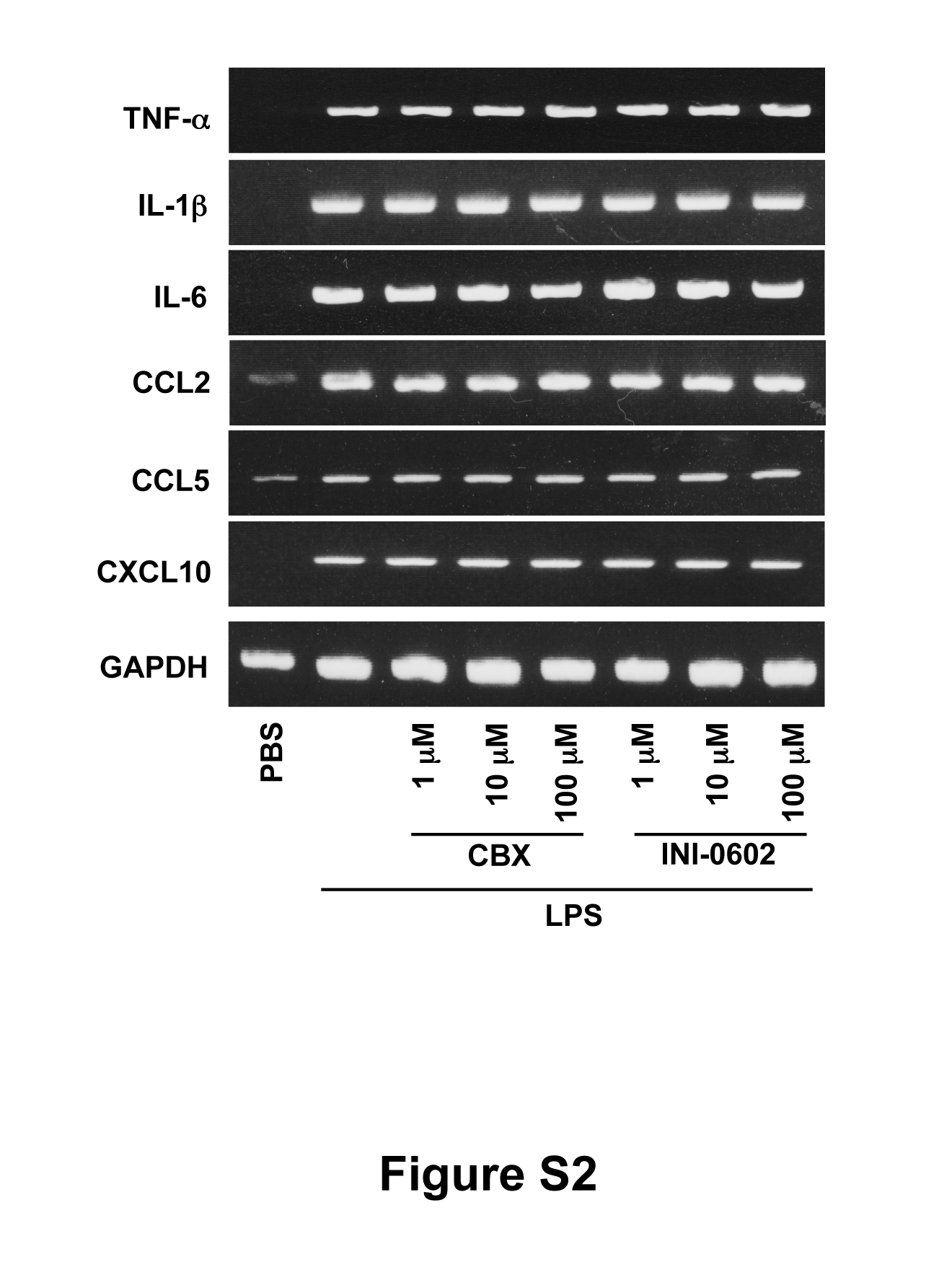

Supplement: Figure S2 — Major cytokine/chemokine expression by microglia treated with gap junction hemichannel blockers. Representative RT-PCR data of major cytokine/chemokine in microglia. Microglia were treated 1 µg/ml LPS for stimulation. To assess drug effects, cells were simultaneously treated with 1–100 µM CBX or INI-0602. Assessments were performed 24 h after treatment. (TIF) [file pone.0021108.s002.tif]

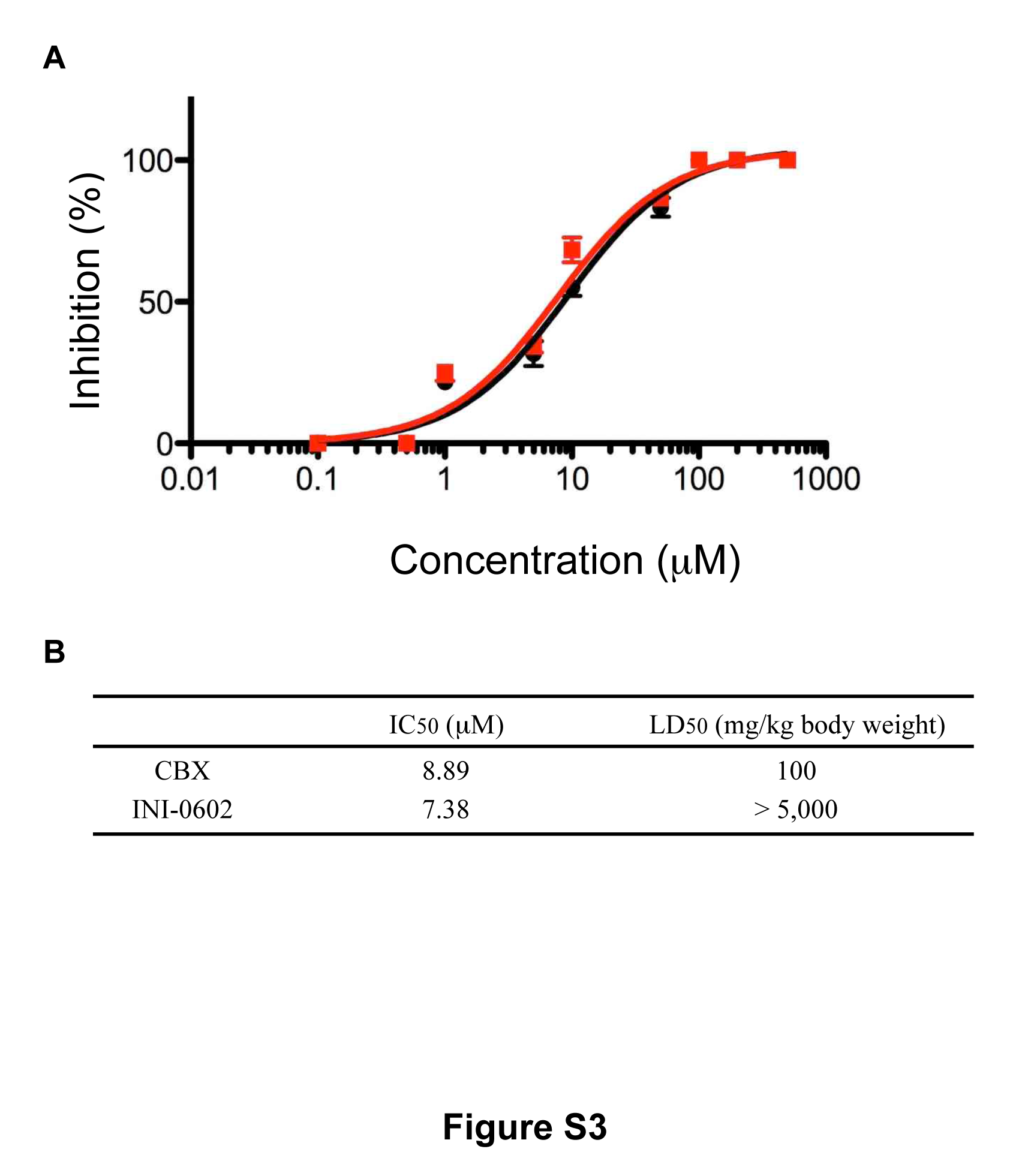

Supplement: Figure S3 — IC50 and LD50 of INI-0602. (A) Inhibition-concentration curves of CBX and INI-0602 for microglial glutamate release. Data represent the means ± SD (n = 6 per group). (B) IC50 in vitro (n = 6 per group) and LD50 in vivo (n = 10 per group) of CBX and INI-0602. (TIF) [file pone.0021108.s003.tif]

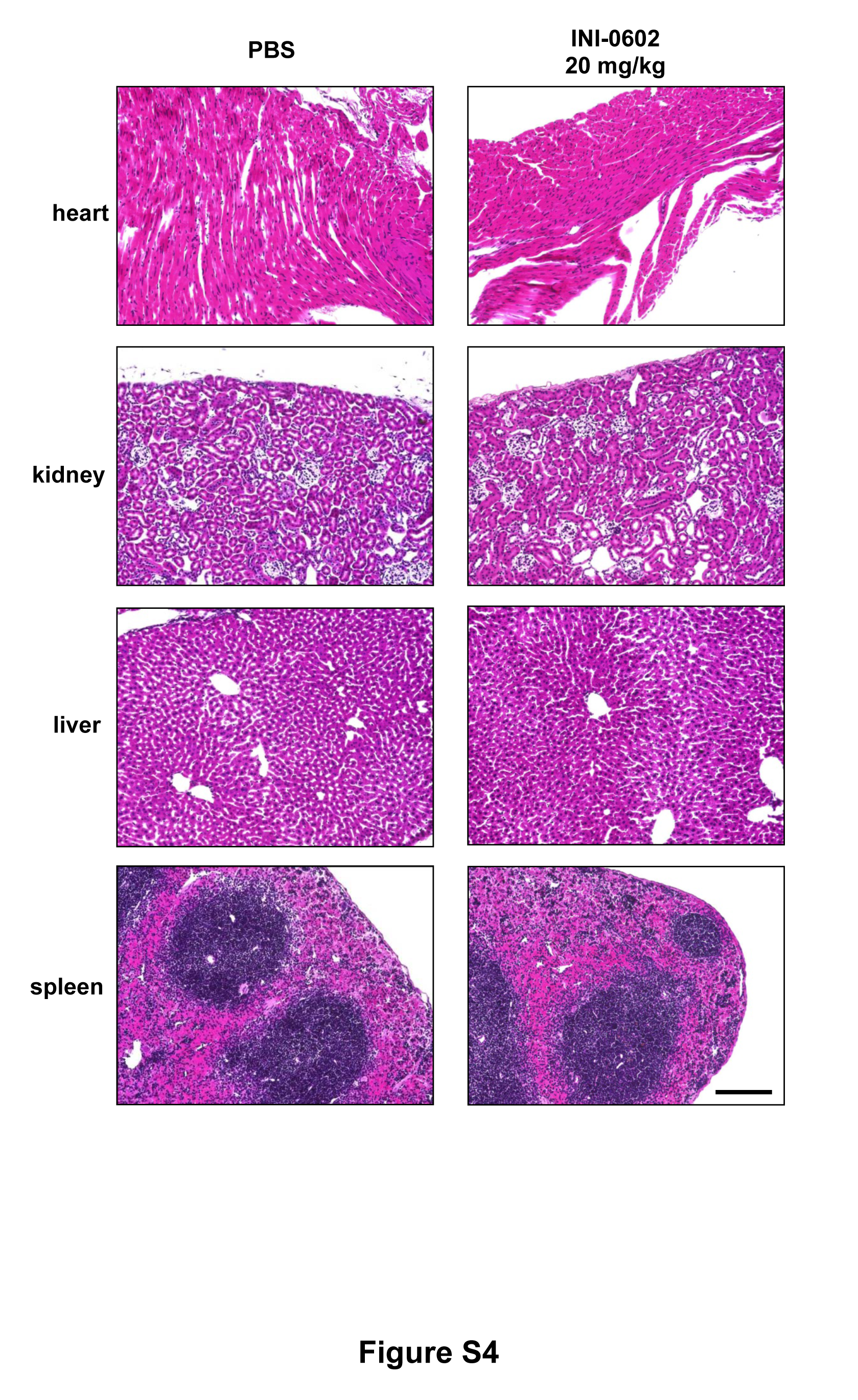

Supplement: Figure S4 — Hematoxylin and eosin staining images of major organs from mice treated with PBS or INI-0602. Representative hematoxylin and eosin staining images of the heart, kidney liver and spleen from 12-week-old C57BL/6J mice treated with PBS or 20 mg/kg INI-0602 every other day for five months. No significant differences were observed. Scale bar, 200 µm. (TIF) [file pone.0021108.s004.tif]

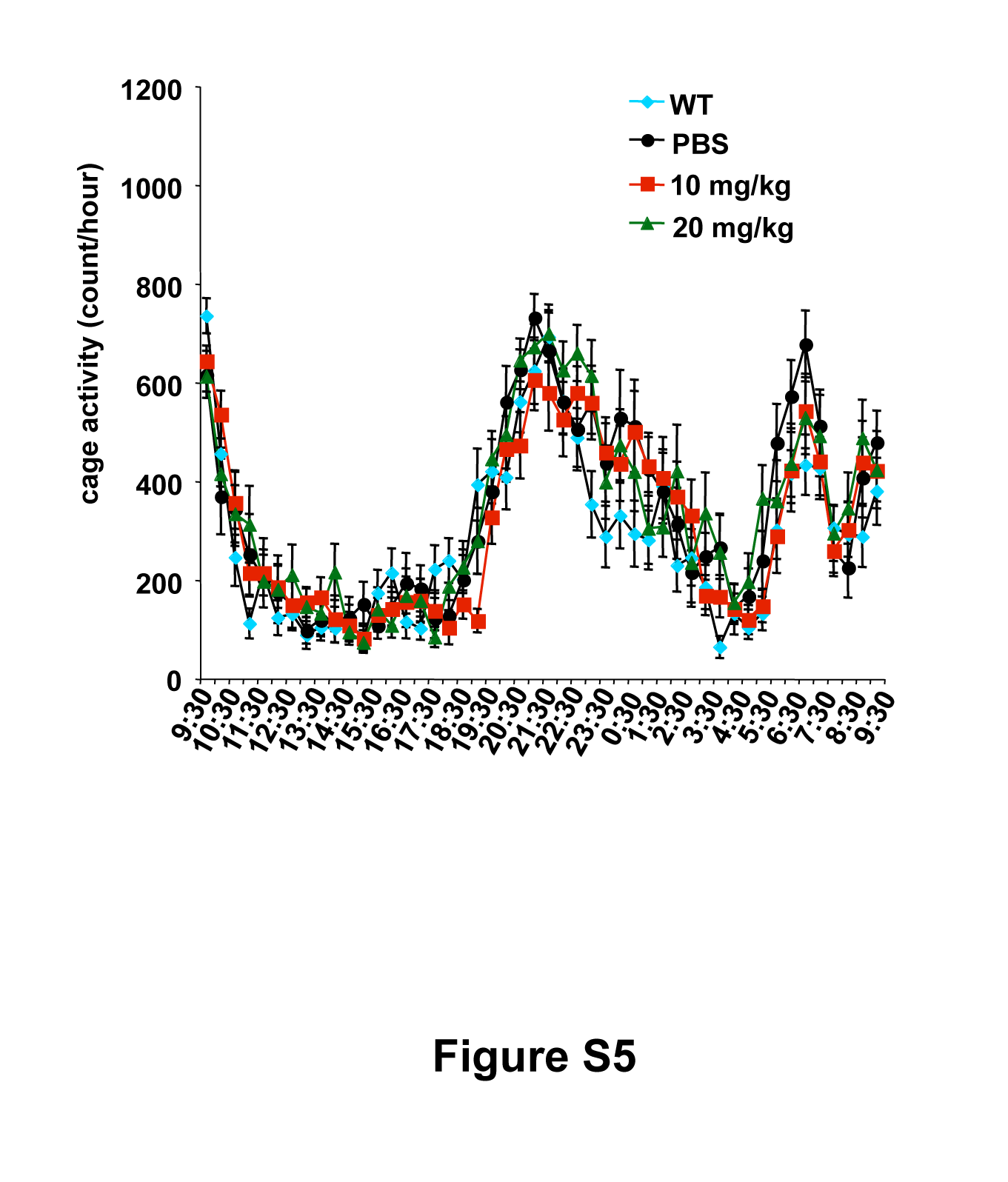

Supplement: Figure S5 — Assessment of 24-h cage activity of mice treated with PBS or INI-0602. Representative 24-h cage activity data of 12-week-old C57BL/6J mice treated with PBS or 20 mg/kg INI-0602 every other day for five months. No significant difference was observed. Data represent the means ± SD (n = 8 per group). (TIF) [file pone.0021108.s005.tif]
