# Supplementary material for: Blockade of Gap Junction Hemichannel Suppresses Disease Progression in Mouse Models of Amyotrophic Lateral Sclerosis and Alzheimer's Disease
Source: PLoS One. 2011 Jun 21;6(6):e21108. doi: 10.1371/journal.pone.0021108 (PMC3119678; doi:10.1371/journal.pone.0021108)
Supplement: Table S1 — Drug concentrations from plasma and brain tissues. Mice were intravenously injected with 20 mg/kg CBX or INI-0602. Drug concentrations were assessed using HPLC/MS. ND, not detected. (DOC) [file pone.0021108.s006.doc]

**Table S1. Drug concentrations from plasma and brain tissues.**

CBX

| **Time (min)** | **plasma (ng/ml)** | **brain (ng/mg tissue)** | **brain/plasma** |
| --- | --- | --- | --- |
| 5 | 21.7 × 10^3^ | ND | 0 |
| 10 | 10.7 × 10^3^ | ND | 0 |
| 15 | 6.35 × 10^3^ | ND | 0 |
| 30 | 2.02 × 10^3^ | ND | 0 |
| 60 | 1.63 × 10^3^ | ND | 0 |
| 180 | 0.20 × 10^3^ | ND | 0 |

INI-0602

| **Time (min)** | **plasma (ng/ml)** | **brain (ng/mg tissue)** | **brain/plasma** |
| --- | --- | --- | --- |
| 5 | 23.5 × 10^3^ | 114 × 10^3^ | 5.0 |
| 10 | 8.62 × 10^3^ | 81.6 × 10^3^ | 9.0 |
| 15 | 6.43 × 10^3^ | 159 × 10^3^ | 25.0 |
| 30 | 1.69 × 10^3^ | 22.4 × 10^3^ | 13.0 |
| 60 | 1.34 × 10^3^ | 18.7 × 10^3^ | 14.0 |
| 180 | 0.20 × 10^3^ | 1.39 × 10^3^ | 7.0 |
