# Supplementary material for: Blockade of Gap Junction Hemichannel Suppresses Disease Progression in Mouse Models of Amyotrophic Lateral Sclerosis and Alzheimer's Disease
Source: PLoS One. 2011 Jun 21;6(6):e21108. doi: 10.1371/journal.pone.0021108 (PMC3119678; doi:10.1371/journal.pone.0021108)
Supplement: Table S2 — Blood analysis of 12-week-old mice treated with PBS or INI-0602. Wild-type C57BL6/J mice were treated with PBS or INI-0602 (5, 10, 20, or 40 mg/kg) every other day for five months. Blood was collected from the inferior aorta under deep anesthesia. Whole blood and serum were analyzed for the items as below with an autoanalyzer (Nagahama Life Science Laboratory, Nagahama, Japan). Data represent the means ± SE (n = 10 per group). (DOC) [file pone.0021108.s007.doc]

**Table S2. Blood analysis of 12-week-old mice treated with PBS or INI-0602.**

|  | **PBS** | **5 mg/kg** | **10 mg/kg** | **20 mg/kg** | **40 mg/kg** |
| --- | --- | --- | --- | --- | --- |
| **white blood cells (× 10^2^/µl)** | 31 ± 2.0 | 25 ± 15 | 27 ± 13 | 30 ± 5.0 | 28 ± 15 |
| **red blood cell (× 10^4^/µl)** | 929 ± 11 | 914 ± 21 | 927 ± 10 | 911 ± 17 | 926 ± 18 |
| **hemoglobin (g/dl)** | 14.1 ± 0.4 | 14.5 ± 0.1 | 14.5 ± 0.5 | 14.2 ± 0.4 | 14.3 ± 0.7 |
| **hematocrit (%)** | 45.1 ± 1.0 | 47.0 ± 1.2 | 44.0 ± 0.5 | 43.7 ± 1.3 | 43.3 ± 1.1 |
| **platelet (× 10^4^/µl)** | 92.3 ± 3.5 | 109.1 ± 10.5 | 97.9 ± 2.1 | 107.0 ± 7.4 | 102.9 ± 9.5 |
| **total protein (g/dl)** | 5.2 ± 0.7 | 5.1 ± 0.4 | 5.2 ± 0.4 | 5.3 ± 0.3 | 5.4 ± 0.6 |
| **creatinine (mg/dl)** | 0.18 ± 0.05 | 0.16 ± 0.02 | 0.15 ± 0.05 | 0.16 ± 0.03 | 0.15 ± 0.03 |
| **uric acid (mg/dl)** | 2.5 ± 0.5 | 2.2 ± 0.4 | 1.9 ± 0.6 | 2.0 ± 0.8 | 2.1 ± 0.7 |
| **Na (mEq/l)** | 157 ± 3.0 | 155 ± 2.0 | 154 ± 3.0 | 155 ± 3.0 | 155 ± 3.0 |
| **K (mEq/l)** | 5.4 ± 0.4 | 4.4 ± 0.5 | 4.6 ± 0.7 | 5.4 ± 0.5 | 4.9 ± 0.6 |
| **Cl (mEq/l)** | 110 ± 6.0 | 105 ± 5.0 | 106 ± 2.0 | 103 ± 5.0 | 106 ± 6.0 |
| **alanine aminotransferase (IU/l)** | 26 ± 3.0 | 33 ± 3.0 | 28 ± 4.0 | 29 ± 5.0 | 25 ± 7.0 |
| **amylase (IU/l)** | 1884 ± 61 | 1786 ± 100 | 1716 ± 125 | 1982 ± 157 | 1834 ± 166 |
| **choline esterase (IU/l)** | 27 ± 3.0 | 28 ± 7.0 | 31 ± 7.0 | 35 ± 6.0 | 34 ± 6.0 |
| **glucose (mg/dl)** | 170 ± 52 | 215 ± 67 | 149 ± 32 | 169 ± 51 | 178 ± 56 |
