# Supplementary material for: Blockade of Gap Junction Hemichannel Suppresses Disease Progression in Mouse Models of Amyotrophic Lateral Sclerosis and Alzheimer's Disease
Source: PLoS One. 2011 Jun 21;6(6):e21108. doi: 10.1371/journal.pone.0021108 (PMC3119678; doi:10.1371/journal.pone.0021108)
Supplement: Table S3 — Urinalysis of 12-week-old mice treated with PBS or INI-0602. Wild-type C57BL6/J mice were treated with PBS or INI-0602 (5, 10, 20, or 40 mg/kg) every other day for five months. Urine was collected from the bladder under deep anesthesia and was immediately assessed using Uropaper III (Eiken Chemical, Tokyo, Japan). Data represent the means ± SE (n = 10 per group). ND, not detected. (DOC) [file pone.0021108.s008.doc]

**Table S3. Urinalysis of 12-week-old mice treated with PBS or INI-0602.**

|  | **PBS** | **5 mg/kg** | **10 mg/kg** | **20 mg/kg** | **40 mg/kg** |
| --- | --- | --- | --- | --- | --- |
| **relative density** | 1.02 ± 0.005 | 1.02 ± 0.005 | 1.02 ± 0.005 | 1.02 ± 0.005 | 1.02 ± 0.005 |
| **pH** | 6 ± 1.0 | 6 ± 1.0 | 6 ± 1.0 | 6 ± 1.0 | 6 ± 1.0 |
| **urobillinogen** | normal | normal | normal | normal | normal |
| **blood** | ND | ND | ND | ND | ND |
| **white blood cells** | ND | ND | ND | ND | ND |
| **protein** | ND | ND | ND | ND | ND |
| **glucose** | ND | ND | ND | ND | ND |
| **ketones** | ND | ND | ND | ND | ND |
| **billirubin** | ND | ND | ND | ND | ND |
| **nitrite** | ND | ND | ND | ND | ND |
